# Absorption and Metabolism of Xanthophylls

**DOI:** 10.3390/md9061024

**Published:** 2011-06-10

**Authors:** Eiichi Kotake-Nara, Akihiko Nagao

**Affiliations:** National Food Research Institute, National Agriculture and Food Research Organization, 2-1-12 Kannondai, Tsukuba, Ibaraki 305-8642, Japan; E-Mail: ekotake@affrc.go.jp

**Keywords:** absorption, bioavailability, carotenoid, metabolism, xanthophyll

## Abstract

Dietary carotenoids, especially xanthophylls, have attracted significant attention because of their characteristic biological activities, including anti-allergic, anti-cancer, and anti-obese actions. Although no less than forty carotenoids are ingested under usual dietary habits, only six carotenoids and their metabolites have been found in human tissues, suggesting selectivity in the intestinal absorption of carotenoids. Recently, facilitated diffusion in addition to simple diffusion has been reported to mediate the intestinal absorption of carotenoids in mammals. The selective absorption of carotenoids may be caused by uptake to the intestinal epithelia by the facilitated diffusion and an unknown excretion to intestinal lumen. It is well known that β-carotene can be metabolized to vitamin A after intestinal absorption of carotenoids, but little is known about the metabolic transformation of non provitamin A xanthophylls. The enzymatic oxidation of the secondary hydroxyl group leading to keto-carotenoids would occur as a common pathway of xanthophyll metabolism in mammals. This paper reviews the absorption and metabolism of xanthophylls by introducing recent advances in this field.

## 1. Introduction

Carotenoids, which are synthesized *de novo* by microorganisms and plants, accumulate in various biological tissues throughout the food chain. More than 700 carotenoids, including the metabolites in animals, are present in nature. Most of the carotenoids contain oxygen functions in the molecules, and these carotenoids are referred to as xanthophylls. In recent years, a great deal of attention has been focused on biological activities of dietary xanthophylls such as lutein, zeaxanthin, β-cryptoxanthin, capsanthin, astaxanthin, and fucoxanthin.

Lutein is one of the major xanthophylls present in green leafy vegetables. Lutein and zeaxanthin are known to selectively accumulate in the macula of the human retina. They have been thought to work as antioxidants [1,2] and as blue light filters [3] to protect the eyes from such oxidative stresses as cigarette smoking and sunlight exposure, which can lead to age-related macular degeneration and cataracts. β-Cryptoxanthin, a major xanthophyll in fruits such as papaya and mandarin orange, was reported to decrease the gene expression of interleukin-1β in mouse macrophage RAW264 cells [4], to promote osteoblastic differentiation of mouse MC3T3 cells [5], and to prevent the decrease of calcium content in the bone of ovariectomized rat [6]. Capsanthin, a major xanthophyll in paprika, was reported to increase high-density lipoprotein (HDL)-cholesterol in rat plasma [7].

**Figure 1:**
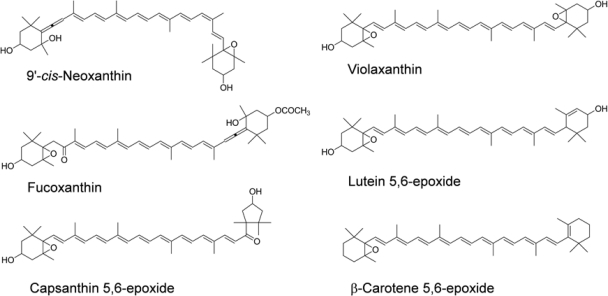
Chemical structures of various epoxy xanthophylls. The geometrical configuration of neoxanthin in nature was recognized as 9′-*cis*.

Astaxanthin and fucoxanthin are abundant in green algae and brown algae, respectively. Numerous studies have reported that astaxanthin has the potential to prevent cancers, diabetes, and inflammatory and cardiovascular diseases [8,9]. Fucoxanthin has been shown to inhibit the growth of various cancer cell lines [10–14] and chemically induced mouse carcinogenesis [15]. Furthermore, the anti-allergic and anti-obese activities of fucoxanthin were recently shown in rodent mast cells [16] and in mice [17], respectively. Neoxanthin, which has a structure similar to that of fucoxanthin, is present in green leafy vegetables. These two xanthophylls have a 5,6-monoepoxide and an allenic bond as the common characteristic functional groups (Figure 1). We found that fucoxanthin and neoxanthin showed the highest inhibitory effect on the proliferation of human prostate cancer cells among the fifteen carotenoids tested [13].

Thus, the characteristic biological activities of several xanthophylls have attracted a great deal of attention. Although no less than forty carotenoids are ingested from common foods, only six major carotenoids (β-carotene, α-carotene, lycopene, β-cryptoxanthin, lutein, and zeaxanthin), their proposed metabolites, and several acyclic carotenes such as phytoene, phytofluene, and ζ-carotene have been found in the plasma of human subjects under usual dietary habits [18,19]. For example, neoxanthin and violaxanthin are ingested together with lutein from green leafy vegetables, but the accumulation of the former two xanthophylls was not confirmed in human plasma [18]. Carotenoids are thought to be selectively absorbed in the human intestine. Moreover, carotenoid accumulations in the biological tissues are known to differ greatly among animal species [20]. However, the mechanisms underlying these phenomena have not been determined.

After intestinal absorption of dietary carotenoids, conversion from β-carotene to vitamin A is well known in animals. In fishes and birds, oxidative and reductive metabolisms of the end group in xanthophylls were also proposed by the identification of the metabolic products, but details as to the mechanism of their metabolic transformation are yet to be elucidated. In mammals, several proposed metabolites of xanthophylls have been detected in the tissues, but the metabolic pathway is still uncertain. It is necessary to reveal the carotenoid metabolism after intestinal absorption in order to elucidate not only the mechanism of the biological activities but also the exact bioavailability. Here, we describe the absorption and metabolism of xanthophylls in mammals.

## 2. Bioaccessibility of Carotenoids

Although xanthophylls have the potential to prevent various degenerative diseases as described above, the bioavailability of carotenoids is lower than that of other fatty components such as α-tocopherol and triacylglycerols [21–24]. The major cause of the low bioavailability is the poor solubility of carotenoids in digestive fluid. Carotenoids must be solubilized in the digestive fluid via several steps before uptake by intestinal epithelial cells can occur [25]. First, carotenoids are released from the food matrix. In some types of food, the matrix interferes with the release of carotenoids. Carotenoids are hardly released from raw vegetables due to the solid structure of the cell walls, but processing and heat treatment of foods accelerate the release of carotenoids by destroying the structures [26]. The released carotenoids must be well dispersed in the gastrointestinal tract. However, the carotenoid dispersion is greatly limited in digestive fluid due to the high hydrophobicity of C40 isoprenoid carbon skeletons. In this step, dietary lipids facilitate the carotenoid dispersion. Carotenoids are dissolved into the dietary lipids and then dispersed as an emulsion in the digestive fluid. The digestion of the dietary lipids in the emulsion progresses with the aid of lipolytic enzymes and bile fluid, and finally the carotenoids are solubilized in the mixed micelle. The mixed micelle consisting of bile acids, phospholipids, cholesterol, fatty acids, and monoacylglycerols has a disk-like shape, in which the outside is surrounded by the bile acids [27]. Carotenoids solubilized in the mixed micelle are thought to become accessible to uptake by the intestinal epithelial cells. Thereby, the bioaccessibility is defined as the ratio of carotenoids solubilized in the mixed micelles to the total carotenoids ingested. The bioaccessibility, dependent on the food matrix, processing, cooking, and structures of carotenoids, is an important factor for bioavailability.

## 3. Intestinal Absorption of Xanthophylls

In addition to the bioaccessibility, carotenoid uptake by intestinal epithelial cells is also a critical factor for the carotenoid bioavailability. Only one part of the accessible carotenoid is taken up by the intestinal epithelial cells and secreted into lymph as chylomicrons for circulating in blood stream. After the chylomicrons are degraded by lipoprotein lipase, carotenoids in chylomicron remnants are taken up by the liver. The carotenoids are stored in liver or resecreted as very-low-density lipoprotein into the blood stream, and then delivered as low-density lipoprotein (LDL). Finally, carotenoids are taken up to tissues through the LDL receptor. Highly hydrophobic carotenoids such as β-carotene and lycopene are localized in the inner part of LDL, while less hydrophobic xanthophylls such as lutein and zeaxanthin are equally distributed to LDL and HDL, and localized in the outer surface area of the lipoprotein particles [28].

The intestinal absorption of carotenoids had been thought to be mediated by simple diffusion [29,30]. To characterize the human intestinal absorption of carotenoids, we compared the uptakes of various carotenoids by human intestinal Caco-2 cells [31]. The carotenoids solubilized at the same concentration in mixed micelles were incubated with the Caco-2 cells. The uptakes were correlated with their lipophilicity, suggesting that simple diffusion mediated the intestinal uptake of the carotenoids. The amounts of fucoxanthin and neoxanthin taken up by the cells were approximately 25% of that of lutein and were the lowest among the eleven carotenoids tested. These results indicated that fucoxanthin and neoxanthin were certainly absorbed in the Caco-2 cells, although the amounts were lower than that of lutein.

In addition to the experiments using Caco-2 cells, we were able to confirm the absorption of these two xanthophylls in mice [32–34]. The xanthophylls solubilized in mixed micelles were orally administrated to male ICR mice. Fucoxanthinol and amarouciaxanthin A derived from fucoxanthin were detected in plasma and the liver [32,33]. A similar result was also reported in rats fed with fucoxanthin [35]. Neoxanthin and neochromes (formed from neoxanthin by intragastric acidity) were detected in plasma and the liver [34]. The plasma concentrations in the mice 2 h after administration of four purified carotenoids (40 nmol) in the independent experiments under almost the same condition were as follows: 36 nM for β-carotene [36]; 10 nM for lutein [36]; 35 nM for neoxanthin (neoxanthin and neochromes) [34] and 50 nM for fucoxanthin (fucoxanthinol and amarouciaxanthin A) [33]. Neoxanthin and fucoxanthin were confirmed to be absorbed at a similar level to those of β-carotene and lutein, and no selectivity for carotenoids tested was found in mice.

In addition to rodents, it has been reported that fucoxanthin is absorbed in other animals such as tunicates [37,38], chicken [39], and aquatic insects [40]. However, fucoxanthin was not absorbed in freshwater fish [40]. East Asian people ingest fucoxanthin from foodstuffs such as sea squirt, sea urchins, mussel, and brown algae. However, no information on the absorption of fucoxanthin in humans has been available. Although neoxanthin and violaxanthin are ingested from green leafy vegetables under usual dietary habits, they were not found in human serum and milk by a detailed analysis of carotenoids [19]. Thus, it has been uncertain whether fucoxanthin and neoxanthin are absorbed in humans.

We reported for the first time the bioavailability of fucoxanthin from edible brown algae (wakame) and of neoxanthin and violaxanthin from spinach in humans [41]. After the daily intake of stir-fried wakame containing 6.1 mg fucoxanthin for 1 week, the concentrations of fucoxanthin and its metabolites in plasma were analyzed by HPLC. Fucoxanthin and amarouciaxanthin A were not detected. Fucoxanthinol was detected, but the plasma concentration was under the quantification limit (1.0 nM). Similar to the case of fucoxanthin, the plasma concentrations of neoxanthin and violaxanthin after the intake of stir-fried spinach were under the quantification limit. On the other hand, both β-carotene and lutein, which were present with these epoxy xanthophylls in the same food matrix of spinach, were increased in the plasma [41], suggesting that little neoxanthin and violaxanthin in spinach were absorbed in humans. In contrast to the case of mice, selective absorption of carotenoids may occur in humans.

The low bioavailability of these epoxy xanthophylls may be caused by their low bioaccessibility from spinach and wakame. However, the bioaccessibility of neoxanthin (neoxanthin and neochromes) from spinach was comparable with that of lutein and was greater than that of β-carotene in *in vitro* digestion study [34]. Similarly, the bioaccessibility of fucoxanthin from wakame was sufficiently high [41]. These results suggested that the bioaccessibility was not a limiting factor of the bioavailability.

The absence of these epoxy xanthophylls in human plasma may be due to the rapid metabolism. However, the concentrations of these epoxy xanthophylls and their metabolites in the plasma were under the quantification limit even shortly after the intake of spinach and wakame [41], indicating that the rapid disappearance might not occur.

It is possible that the level of these epoxy xanthophylls in plasma were estimated to be low due to unknown metabolic transformation such as hydrolysis of epoxide or formation of conjugates by detoxification enzymes after the intestinal uptake. For instance, fucoxanthinol 3′-sulphate found in the egg yolk of hens fed with seaweed meal [39] might be formed from fucoxanthin in humans.

The dietary water-soluble fibers, alginates in wakame may be associated with the low bioavailability of fucoxanthin from wakame in humans, because dietary water-soluble fibers inhibited the β-carotene and lutein uptake by Caco-2 cells [42]. Thus, it is necessary to reveal the bioavailability of isolated carotenoid to avoid the influence of the food matrix.

There are several reports on the bioavailability of epoxy xanthophylls in the purified preparations and the oleoresins in human subjects. Oleoresin, which is extracted from plant materials, does not contain dietary fibers and any other polar substances. Capsanthin 5,6-epoxide and violaxanthin were not detected in chylomicron after ingestion of paprika oleoresin containing these epoxy xanthophylls [43]. However, 9-*cis* zeaxanthin, which was present at a lower amount than epoxy xanthophylls in paprika oleoresin, was found in chylomicron [43]. This result suggested that little capsanthin 5,6-epoxide and violaxanthin in paprika were absorbed in humans. Moreover, after a single oral dose of purified violaxanthin or lutein 5,6-epoxide suspended in corn oil, the two epoxy xanthophylls were not detected in the plasma [44]. In contrast, after an oral dose of purified β-carotene 5,6-epoxide (9.1 μmol) suspended in corn oil, the plasma concentration reached 2.29 μM [45]. Considering these experimental results with the oleoresin and purified xanthophylls, little epoxy xanthophylls that have higher polarity than β-carotene 5,6-epoxide would be absorbed by humans, consistent with the results of our human study using spinach and wakame. The chemical structures of these epoxy xanthophylls are shown in Figure 1.

To summarize the intestinal absorption of carotenoids, little of highly polar epoxy xanthophylls such as neoxanthin and violaxanthin were absorbed in humans independent of the food matrix. Fucoxanthin was absorbed in mice and several other animals, but not in humans and freshwater fishes. A selective absorption mechanism for carotenoids would be present in humans, but not in mice. Moreover, the selectivity in the intestinal absorption of carotenoids appears to differ among animal species.

## 4. Mechanisms of the Intestinal Absorption

The selective absorption for carotenoids in humans cannot be explained by the simple diffusion mechanism alone. On the other hand, recent studies have suggested that the carotenoid uptake is partly mediated by facilitated diffusion [46–53]. For example, the ratio of the uptake mediated by scavenger receptor class B type 1 (SR-B1) to the total uptake of carotenoids in Caco-2 cells was as follows: 50% for β-carotene; 20% for β-cryptoxanthin and 7% for lutein/zeaxanthin [53]. The efficiency of β-carotene absorption was remarkably reduced in SR-B1 knockout mice [54]. The physiological relevance of SR-B1 as an mediator of intestinal uptake for provitamin A carotenoids was indicated by the report that retinoic acid and the intestinal transcription factor ISX regulated expressions of both SR-B1 and β-carotene-15,15′-oxygenase (BCO1), an enzyme responsible for vitamin A production [55]. The facilitated diffusion may cause the selective absorption of carotenoids in humans. However, even if SR-B1 does not mediate intestinal uptake of the highly polar epoxy xanthophylls, they can pass across membranes via the simple diffusion pathway. Thus, these absorption mechanisms could not account for the strict selectivity that was observed in humans. The strict selective absorption might occur if most parts of the highly polar epoxy xanthophylls taken up by intestinal epithelial cells were excreted back into intestinal lumen.

The ATP-binding cassette (ABC) transporters such as ABCG5 and ABCG8 are well known to mediate the excretion of dietary phytosterols [56,57]. Although phytosterols such as β-sitosterol and campesterol are ingested from vegetables, grains, and cooking oils, the serum concentrations of the phytosterols are much lower than that of cholesterol in mammals [56,57]. Interestingly, ABCG5 polymorphism was suggested to be associated with the lutein bioavailability from egg in human subjects [58]. ABCG5 may excrete lutein and highly polar epoxy xanthophylls to intestinal lumen.

**Figure 2:**
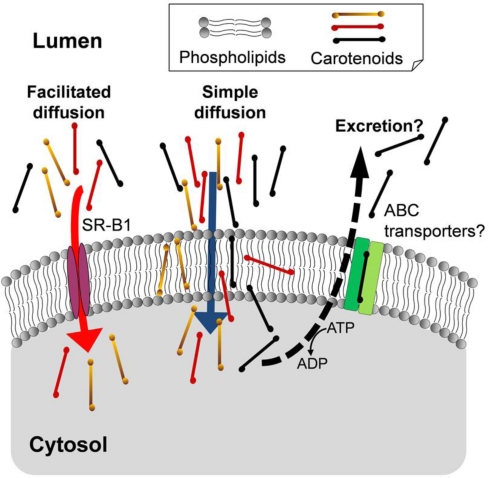
Proposed mechanisms of selectivity in the intestinal absorption of the dietary carotenoids.

Multi-drug resistance 1 (MDR1, ABCB1) is well known as a major efflux pump for lipid-soluble compounds. As the affinity of substrates for MDR1 has been suggested to be related to their polarity [59], the highly polar xanthophylls may be excreted by MDR1. Carotenoids were evaluated for a substrate of MDR1 expressed in certain cancer cells. Neoxanthin and violaxanthin, compared with other carotenoids tested, showed higher affinity for transfected-human MDR1 in mouse lymphoma L1210 cells [60], but similar results were not found in several human breast and colon cancer cell lines [61,62]. Further study is required to confirm the involvement of MDR1 in the excretion of carotenoids in intestinal cells. Thus, the selectivity in the intestinal absorption of carotenoids in humans is likely to be caused by these proteins that mediate uptake and excretion (Figure 2). The specificity of these proteins would cause the differences in the intestinal absorption of carotenoids among animal species.

## 5. Metabolism of Xanthophylls in Mammals

It is necessary to explore the metabolism of carotenoids after intestinal absorption in order to elucidate the mechanism of their biological activities, and to achieve safe and effective applications to human subjects. Although β-carotene is known to be metabolized to vitamin A through action of BCO1, little is known about the metabolic transformation of non provitamin A xanthophylls in mammals.

**Figure 3:**
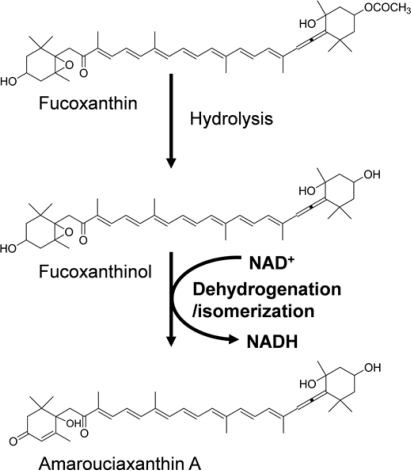
Proposed metabolic transformation of fucoxanthin.

Recently, we obtained evidence that the oxidative transformation of fucoxanthin and lutein to keto-carotenoids occurred in mammals. Fucoxanthinol and amarouciaxanthin A were found in the plasma and liver of mice fed with fucoxanthin, whereas fucoxanthin itself was not detected [32,33]. Fucoxanthinol was hydrolyzed from fucoxanthin in the intestinal tract, circulated in the body, and then oxidatively converted into amarouciaxanthin A (Figure 3). The conversion of fucoxanthinol into amarouciaxanthin A was also found to occur in human hepatoma HepG2 cells. Moreover, we found for the first time that the oxidative conversion was mediated in mouse liver microsomal fractions and required NAD^+^ as a cofactor, demonstrating the metabolic conversion of the 3-hydroxyl end group in xanthophylls at the level of enzyme reaction in animals [33].

**Figure 4:**
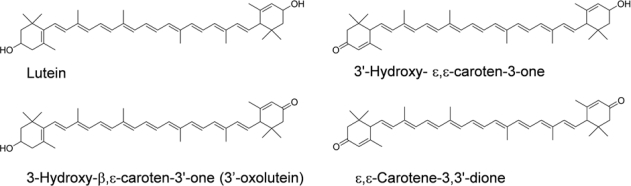
Chemical structures of lutein and its metabolites.

Several proposed metabolites of lutein, as shown in Figure 4, were previously known to be present in such human tissues as plasma, milk, liver, and retina [18,63–66]. Moreover, we found a remarkable accumulation of metabolites in mice fed with lutein [67]. 3′-Hydroxy-ɛ,ɛ-caroten-3-one and lutein were the predominant carotenoids in the plasma, liver, kidney, and adipose, accompanied by ɛ,ɛ-carotene-3,3′-dione, indicating that mice actively convert lutein to keto-carotenoids by oxidizing the secondary hydroxyl group. However, 3-hydroxy-β,ɛ-caroten-3′-one (3′-oxolutein), the major metabolite of lutein in human plasma [67] and the retina [64], was not detected in the tissues of the mice.

These metabolites would be formed by the same enzyme that mediated the conversion of fucoxanthinol to amarouciaxanthin A. The combined level of the lutein metabolites in the liver of the mice was 72.4% of the total (intact lutein and the metabolites) [67]. This indicates that quantification of the metabolites is necessary to estimate the lutein bioavailability. Moreover, intact lutein and the metabolites may differ in their biological activities. Differences among lutein and its metabolites as antioxidants and blue light filters deserved further study.

Similar to the case of lutein in mice, the oxidative metabolism of the other xanthophylls was reported to occur in human subjects. After the ingestion of paprika juice containing capsanthin as a major xanthophyll, capsanthon in addition to capsanthin was found in the plasma [68]. Capsanthon may be formed from capsanthin by the oxidation of the 3′-hydroxyl group to the 3′-keto group. After an oral dose of 4,4′-dimethoxy-β-carotene in peanut oil, both 4-keto-β-carotene and canthaxanthin were found in the plasma [69]. These studies certainly indicate that humans have potential metabolic activity for the oxidation of secondary hydroxyl groups in various xanthophylls.

In human tissues, other metabolites of lutein were detected. 3′-Epilutein might be formed by a back reduction of 3′-oxolutein that was produced from lutein [64]. *meso*-Zeaxanthin, which is detected in the retina only, might be formed by double bond migration from lutein [64]. The dehydration products of lutein, 3-hydroxy-3′,4′-didehydro-β,γ-carotene and 3-hydroxy-2′,3′-didehydro-β,ɛ-carotene [19] were thought to be formed non-enzymatically under acidic conditions of stomach [70,71].

Recent studies have indicated the cleavage reaction of xanthophylls occurred in mammals. BCO1 catalyzes the central cleavage of provitamin A carotenoids, while β-carotene 9′,10′-oxygenase (BCO2) expressed *in vitro* can cleave a double bond at C-9′ and C-10′ of β-carotene, lycopene and xanthopylls [72–74]. Nonsense mutation of BCO2 was found to be associated with a yellow fat phenotype in sheep, in which xanthophylls were accumulated in adipose tissues [75]. The BCO2 knockout mice fed with lutein remarkably accumulated lutein metabolites, compared with the wild-type mice [76]. BCO2 might reduce the accumulation of xanthophylls by converting to smaller molecules, although the cleavage products and their further metabolites have not been detected in animal tissues yet. Thus, in addition to oxidation of secondary hydroxyl group in xanthophylls, the cleavage reaction of carbon skeleton by BCO2 would be also a major metabolic transformation of xanthophylls in mammals.

## 6. Conclusions

Various carotenoids, in particular, xanthophylls are ingested under usual dietary habits. However, carotenoids accumulated in human tissues are limited, suggesting selectivity in the intestinal absorption and different metabolic fates of carotenoids. The responses to the feeding of highly polar xanthophylls indicated that, for humans, intestinal absorption would be strictly selective in comparison with mice. The selectivity and its differences among animal species cannot be explained by simple diffusion mechanism alone. Instead, facilitated diffusion via SR-B1 and an unknown excretion to luminal side might cause the selectivity. After intestinal absorption of xanthophylls, the enzymatic oxidation of the secondary hydroxyl group leading to keto-carotenoids would occur as a common pathway of xanthophyll metabolism in mammals. We have no knowledge about the relation of these metabolites to the biological activities of parental xanthophylls. The potential biological activities of xanthophyll metabolites and their further metabolic fates warrant future studies with respect to the beneficial effects of xanthophylls on human health.
